# From therapeutic landscape perception to revisit intention: the mediating role of restorative experience in Palandöken Urban Forest

**DOI:** 10.3389/fpubh.2026.1946982

**Published:** 2026-10-13

**Authors:** Murat Göral

**Affiliations:** Department of Tourism and Hotel Management, Bingöl University, Bingöl, Türkiye

**Keywords:** Palandöken, PLS-SEM, restorative experience, revisit intention, therapeutic landscape, urban forest, visitor satisfaction

## Abstract

**Purpose:**

This study examines the mediating role of restorative experience in the associations between therapeutic landscape perception, visitor satisfaction, and revisit intention in Palandöken Urban Forest.

**Methodology:**

A quantitative, cross-sectional survey design was employed. Data were collected from 413 visitors through face-to-face questionnaires and analyzed using PLS-SEM in SmartPLS 4. Therapeutic landscape perception was measured through the natural, aesthetic, and social landscape dimensions. Because all focal variables were measured at the same time, structural paths and indirect effects are interpreted as associations consistent with the proposed model rather than as evidence of temporal or causal processes.

**Findings:**

All dimensions of therapeutic landscape perception showed significant positive associations with restorative experience. Restorative experience was positively associated with both visitor satisfaction and revisit intention, while satisfaction was also positively associated with revisit intention. The model explained 48.7% of the variance in restorative experience, 54.7% of the variance in satisfaction, and 52.8% of the variance in revisit intention. Significant indirect effects were observed between the dimensions of therapeutic landscape perception and both visitor outcomes through restorative experience, consistent with the proposed partial-mediation pattern. The direct associations of the three landscape dimensions with revisit intention were statistically significant but very small in substantive magnitude (*f*^2^ = 0.010–0.017).

**Originality/value:**

Rather than proposing a new theoretical model, this study provides an incremental theoretical refinement by distinguishing therapeutic landscape perception as an appraisal of environmental qualities from restorative experience as a separate self-reported experiential state. The findings indicate that direct associations between landscape dimensions and revisit intention are weak in magnitude, whereas restorative experience and satisfaction occupy more prominent positions in the proposed pathway. The public-health relevance lies in clarifying how perceived therapeutic qualities and self-reported restoration are associated with continued recreational engagement with an accessible urban green space; satisfaction and revisit intention are treated as tourism/recreation outcomes, not as health outcomes.

## 1 Introduction

Accelerating urbanization, the increasingly demanding rhythm of everyday life, and individuals’ growing exposure to psychological strain have made it necessary to reconsider the functions of urban green spaces not only in terms of recreation but also in relation to mental restoration, emotional relief, and wellbeing (1–3). Urban and peri-urban nature-based areas have become important public resources associated with stress relief, attentional restoration, and psychological recovery (4, 5). Research in environmental psychology, public health, and health geography shows that contact with natural environments is associated with mental health, positive affect, physical activity, social interaction, and overall wellbeing (6–12). Urban forests should therefore be considered not only in terms of their physical environmental quality and recreational opportunities but also in relation to the therapeutic qualities perceived by visitors and the self-reported restorative experiences associated with their use.

Urban forests form transitional spaces between the built environment and the natural landscape, offering visitors opportunities to step away from everyday life, connect with nature, and report restorative experiences (13). The natural character, open views, silence, clean air, spatial spaciousness, and social atmosphere of these areas may extend the visitor experience beyond physical recreation (13). Recent studies show that environmental qualities in urban forests and urban green spaces are associated with visitor satisfaction, positive behavioral intentions, and revisit intention (14, 15). However, explaining visitor behavior solely through environmental characteristics or service quality is overly simplistic. Perceived relief, restoration, and wellbeing associated with a setting are also relevant to how visitors evaluate that place.

In this context, the therapeutic landscape perspective provides a theoretical basis for examining relationships between nature-based recreational spaces, perceived restoration, and wellbeing. Gesler (16) introduced the concept within health geography, proposing that places may promote health and wellbeing through their physical, social, symbolic, and cultural characteristics. Researchers have subsequently extended this perspective from clinical or sacred healing sites to everyday living environments, tourism destinations, coastal areas, rural settings, and urban green spaces (17–19). Accordingly, identifying a therapeutic landscape involves more than evaluating its beauty or utility; it also requires considering how the landscape is associated with relaxation, security, escape, belonging, restoration, and wellbeing.

Restorative experience is treated in this study as a distinct self-reported experiential construct that may help explain the association between therapeutic landscape perception and visitor outcomes. Therapeutic landscape perception captures visitors’ appraisal of the healing- and wellbeing-supporting qualities of an environment, whereas restorative experience refers to reported mental relief, attentional renewal, and a sense of being away from everyday pressures. Attention Restoration Theory proposes that natural environments can support restoration through the qualities of “being away,” “fascination,” “extent,” and “compatibility.” Stress Reduction Theory, in turn, proposes that natural environments may support psychological recovery by increasing positive affect and reducing stress responses (8, 9, 20, 21). Within the present cross-sectional design, these theories provide a conceptual rationale for examining associations among perceived landscape qualities, self-reported restorative experience, and visitor outcomes, rather than establishing temporal or causal effects.

In tourism and recreation research, satisfaction and revisit intention are among the outcome variables most commonly used to explain visitor behavior. Satisfaction refers to visitors’ overall evaluation of an experience, whereas revisit intention refers to their tendency to choose the same area again in the future (22–28). These variables are tourism and recreation outcomes rather than health outcomes. In nature-based and health-supportive recreational settings, however, they can help explain whether visitors positively evaluate and intend to reuse environments they perceive as restorative. Recent studies show that perceived restorative environments, flow experiences, hedonic and eudaimonic wellbeing, and perceived destination restorative qualities are associated with post-visit behavior, loyalty, and revisit intention (29–31).

A more specific conceptual issue remains insufficiently clarified in the literature. Therapeutic landscape perception and restorative experience are closely related but theoretically distinct constructs: the former concerns how visitors evaluate the therapeutic qualities of an environment, whereas the latter concerns the restorative experience they report in relation to that environment. Existing studies have not consistently examined this distinction together with satisfaction and revisit intention within the same empirical framework. Accordingly, the contribution of the present study is not the introduction of a new theoretical model, but an incremental clarification of how environmental appraisal, self-reported restorative experience, and recreation-related outcomes are associated with one another. In particular, the model makes it possible to examine whether restorative experience provides an additional explanatory layer between therapeutic landscape perception and visitor outcomes beyond the direct associations between environmental perceptions and behavioral intentions.

Palandöken Urban Forest provides a useful research setting for this purpose. Located in the mountainous environment of Erzurum, the site differs from typical urban green spaces through its high altitude, forested landscape, expansive views, seasonal variation, accessibility from the city center, and recreational potential. Its mountainous landscape, distinctive climate, and perceived distance from urban life may be associated with visitors’ therapeutic landscape perceptions and restorative experiences. The site therefore offers a context in which to examine how an accessible urban forest is perceived simultaneously as a recreational setting and as an environment associated with self-reported restoration.

This study aims to investigate the statistical mediating role of restorative experience in the associations between therapeutic landscape perception, visitor satisfaction, and revisit intention, using Palandöken Urban Forest as a case study. Therapeutic landscape perception is examined through its natural, aesthetic, and social dimensions; restorative experience is treated as a separate self-reported experiential state; and satisfaction and revisit intention are treated as post-visit tourism/recreation outcomes. The public-health relevance of the study lies not in treating satisfaction or revisit intention as indicators of health improvement, but in examining how perceived therapeutic qualities and self-reported restoration are associated with visitors’ evaluations and intentions to reuse an accessible public green space.

## 2 Theoretical framework and hypothesis development

Recent tourism research has emphasized that associations between environmental characteristics and visitor behavior may be better understood when internal experiential states such as satisfaction, wellbeing, flow experience, destination image, and restorative experience are considered (15, 29, 31). Research in health-tourism settings further indicates that aesthetic and escapist experience dimensions are positively associated with tourist inspiration, which in turn is associated with tourist engagement (32). Evidence from a hedonic consumption context likewise indicates that inspiration and pleasure are associated with subsequent behavioral intentions (33). This perspective provides the theoretical basis for examining whether therapeutic landscape perception is associated with revisit intention both directly and indirectly through self-reported restorative experience, without assuming temporal or causal ordering from the present cross-sectional data.

### 2.1 Therapeutic landscape perception and restorative experience

Therapeutic landscape perception refers to visitors’ evaluation of an environment as possessing healing, relaxing, and wellbeing-supporting qualities (34). Restorative experience, by contrast, refers to self-reported mental relief, attentional renewal, and a sense of being away from everyday pressures that is conceptually related to such environmental evaluations (20). The two concepts are therefore distinguished analytically: therapeutic landscape perception concerns appraisal of the environment, whereas restorative experience concerns a reported experiential state associated with that appraisal.

This proposed relationship is supported conceptually by the restorative environment literature. Attention Restoration Theory argues that natural environments may support restoration through the qualities of “being away,” “fascination,” “extent,” and “compatibility” (9). Stress Reduction Theory similarly proposes that natural environments may be associated with psychological relaxation through positive affect and reduced stress responses (21). Therapeutic landscape qualities such as naturalness, landscape quality, silence, clean air, spatial organization, safety, and social atmosphere may therefore be positively associated with visitors’ restorative experiences.

Li and He (29) found that perceived restorative environments were associated with tourists’ flow experiences and post-visit behavior. Feng et al. (35) reported associations between natural, social, and aesthetic dimensions of therapeutic landscapes and tourists’ health perceptions. Zhu et al. (31) found that perceived destination restorative qualities were associated with hedonic and eudaimonic experiences. Together, these findings suggest that an urban forest perceived as possessing strong therapeutic qualities may also be associated with stronger self-reported restorative experiences.

*H1a*: Natural landscape perception is positively associated with restorative experience.

*H1b*: Aesthetic landscape perception is positively associated with restorative experience.

*H1c*: Social landscape perception is positively associated with restorative experience.

### 2.2 Therapeutic landscape perception and visitor satisfaction

Satisfaction refers to visitors’ overall evaluation of their experience. In nature-based recreational areas, satisfaction does not depend solely on functional elements such as service quality, physical facilities, or accessibility. Feelings of peace, safety, relaxation, aesthetic pleasure, and perceived wellbeing within the environment may also be associated with satisfaction. An environment perceived as therapeutic is therefore expected to be positively associated with the overall evaluation of the visit.

In the context of urban forests, Yao et al. (15) showed that the recreational environment is associated with visitor satisfaction through the spatial-physical environment, service experience, and regional and cultural characteristics. In wellness tourism, Seow et al. (36) found that satisfaction is particularly associated with emotional and experiential values. Similarly, Vapur (37), in a study of Aydos Forest, reported a strong positive relationship between psychological benefits derived from nature-based recreation and visitor satisfaction.

*H2a*: Natural landscape perception is positively associated with visitor satisfaction.

*H2b*: Aesthetic landscape perception is positively associated with visitor satisfaction.

*H2c*: Social landscape perception is positively associated with visitor satisfaction.

### 2.3 Therapeutic landscape perception and revisit intention

Revisit intention refers to visitors’ tendency to choose the same destination or recreational area again in the future. When visitors perceive an area as therapeutic, it may become a meaningful recreational resource associated with personal wellbeing. As visitors view a place as stress-reducing, mentally restorative, and emotionally calming, stronger revisit intention may also be reported. This relationship is particularly relevant in nature-based and urban-forest settings.

Li and He (29) indicated that perceived restorative environments are associated with post-visit behavioral tendencies through novelty, escape, fascination, and compatibility. Yao et al. (15) further demonstrated associations between the recreational environment of an urban forest and positive behavioral intentions such as revisiting and recommending. These findings suggest that therapeutic landscape perception may be positively associated with revisit intention.

*H3a*: Natural landscape perception is positively associated with revisit intention.

*H3b*: Aesthetic landscape perception is positively associated with revisit intention.

*H3c*: Social landscape perception is positively associated with revisit intention.

### 2.4 Restorative experience and visitor satisfaction

Restorative experience refers to visitors’ self-reported sense of mental and emotional renewal during their interaction with a place. It can therefore be considered a theoretically relevant correlate of satisfaction. Particularly in nature-based recreational areas, visitor satisfaction may be related not only to the physical quality of the environment but also to reported relaxation, relief, and renewal during the experience.

Zhu et al. (31) showed that perceived destination restorative qualities are associated with hedonic and eudaimonic experiences and loyalty. Wang et al. (38) found that perceived restorative destination environments are associated with willingness to pay for environmental protection through eudaimonic wellbeing and destination image. In the context of urban forests, Vapur (37) reported a strong relationship between the psychological benefits of recreation and visitor satisfaction.

*H4*: Restorative experience is positively associated with visitor satisfaction.

### 2.5 Restorative experience and revisit intention

Restorative experience is positioned as an important experiential component in the proposed pathway to revisit intention. The decision to revisit may be associated not only with a favorable evaluation of a previous experience but also with the desire to experience similar perceived restoration again. When visitors report mental relaxation, emotional calmness, and a sense of being away from everyday life in an urban forest, they may also report stronger intentions to return.

Li and He (29) showed that perceived restorative environments are associated with post-visit behavior through flow experience. Zhu et al. (31) found that restorative destination qualities are associated with loyalty through hedonic and eudaimonic experiences. Studies in nature-based tourism also indicate associations between subjective wellbeing and revisit intention.

*H5*: Restorative experience is positively associated with revisit intention.

### 2.6 Visitor satisfaction and revisit intention

Satisfaction is one of the most established correlates of revisit intention in the tourism literature. Visitors who are satisfied with their experience are more likely to report an intention to choose the same destination again and to develop positive behavioral intentions (22–24). In this study, satisfaction is treated as an evaluative tourism/recreation outcome associated with revisit intention, not as a health outcome.

Yao et al. (15) showed that, in urban forests, satisfaction is positively associated with post-visit behavioral intention and statistically mediates the relationship between environmental perception and behavioral intention. Seow et al. (36) reported that tourist satisfaction in wellness tourism is associated with revisit intention and electronic word-of-mouth communication.

*H6*: Visitor satisfaction is positively associated with revisit intention.

### 2.7 The mediating role of restorative experience

The proposed model examines whether the associations of therapeutic landscape perception with visitor satisfaction and revisit intention include statistically significant indirect components through restorative experience. Therapeutic landscape perception represents an environmental evaluation (17), whereas restorative experience represents a distinct self-reported experiential state (39). The mediation analysis therefore tests an indirect association consistent with the proposed theoretical pathway; given the cross-sectional design, it does not establish temporal or causal mediation.

The Stimulus–Organism–Response (S-O-R) model provides the conceptual logic for the proposed relationships among the study variables. Originally developed by Mehrabian and Russell (40), the model explains how external environmental stimuli (S) are associated with individuals’ internal cognitive and affective states (O), which are subsequently related to behavioral or evaluative responses (R). In the context of the present study, therapeutic landscape perception represents the stimulus component because it reflects visitors’ appraisal of the therapeutic qualities of the surrounding environment. Restorative experience represents the organism component, as it captures visitors’ internal experiential state, including psychological relief, attentional renewal, and a sense of being away from everyday demands. Visitor satisfaction and revisit intention represent response components, reflecting evaluative and behavioral outcomes associated with these internal experiences. Accordingly, the S-O-R framework provides a theoretical basis for examining whether therapeutic landscape perception is associated with satisfaction and revisit intention both directly and indirectly through restorative experience. Recent studies report comparable indirect patterns. Li and He (29) showed that flow experience statistically mediates the association between perceived restorative environments and post-visit behavior. Yao et al. (15) supported a statistical mediating role of satisfaction in the association between the urban-forest environment and behavioral intention. Zhu et al. (31) found that the relationship between restorative qualities and loyalty is associated with wellbeing experiences.

*H7a*: Restorative experience statistically mediates the association between natural landscape perception and visitor satisfaction.

*H7b*: Restorative experience statistically mediates the association between aesthetic landscape perception and visitor satisfaction.

*H7c*: Restorative experience statistically mediates the association between social landscape perception and visitor satisfaction.

*H8a*: Restorative experience statistically mediates the association between natural landscape perception and revisit intention.

*H8b*: Restorative experience statistically mediates the association between aesthetic landscape perception and revisit intention.

*H8c*: Restorative experience statistically mediates the association between social landscape perception and revisit intention.

The conceptual model of the study, developed within the S-O-R framework, is presented in Figure 1. The model addresses therapeutic landscape perception through natural, aesthetic, and social landscape dimensions and positions restorative experience as the proposed intervening experiential construct associated with visitor satisfaction and revisit intention.

**Figure 1:**
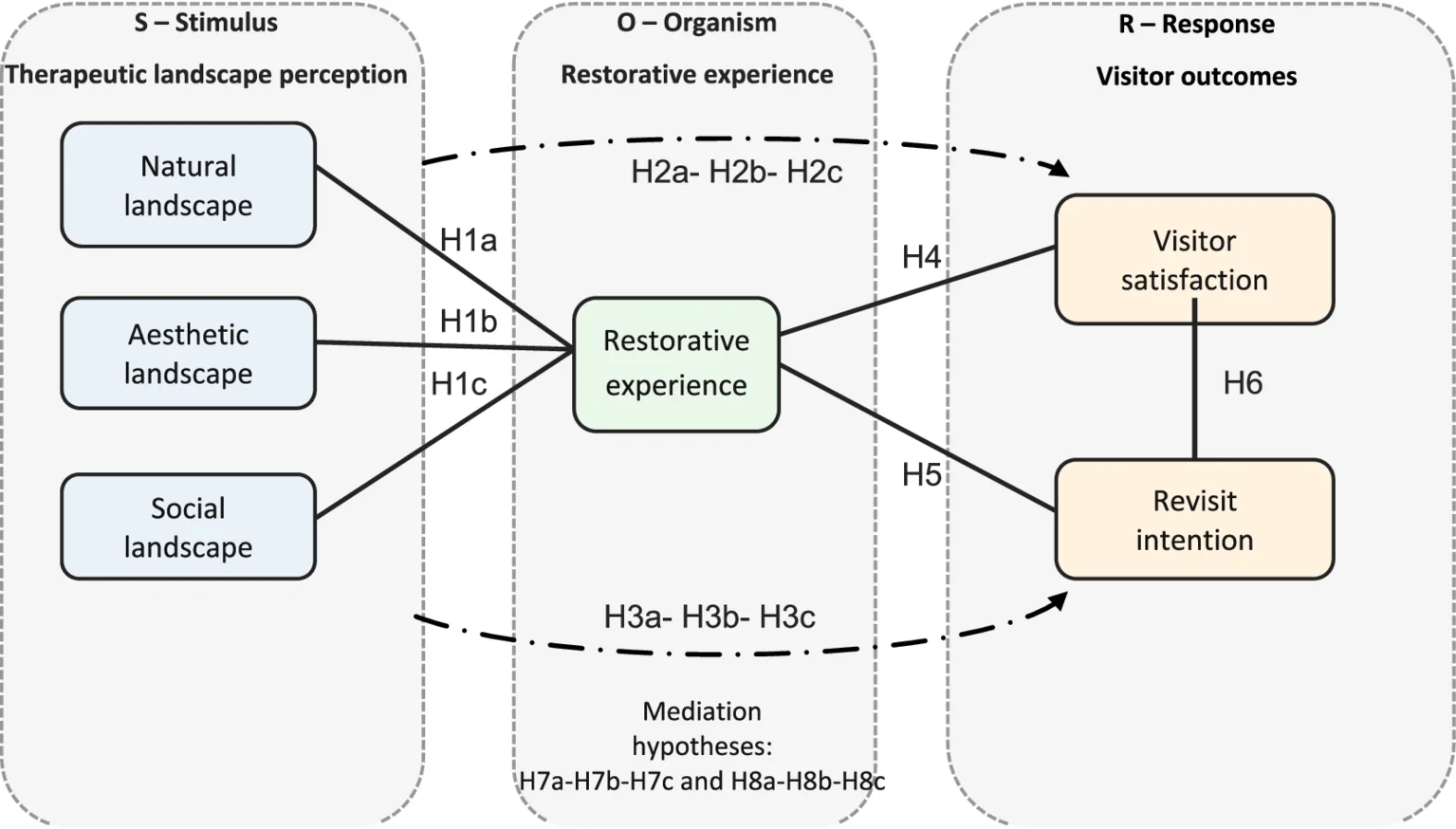
Research model.

H1a–H1c indicate the proposed direct associations of the natural, aesthetic, and social landscape dimensions with restorative experience; H2a–H2c and H3a–H3c indicate their direct associations with visitor satisfaction and revisit intention, respectively; H4 and H5 indicate the associations of restorative experience with satisfaction and revisit intention; H6 indicates the association between satisfaction and revisit intention. H7a–H7c and H8a–H8c denote the proposed indirect paths through restorative experience.

## 3 Methodology

### 3.1 Research design

This study employed a quantitative, cross-sectional, and explanatory research design to examine associations among therapeutic landscape perception, restorative experience, satisfaction, and revisit intention among visitors to Palandöken Urban Forest. In the model, therapeutic landscape perception was specified as the exogenous construct, restorative experience as the statistical mediator, and satisfaction and revisit intention as outcome constructs. The theoretical model proposes an ordered pathway among these variables; however, because all focal variables were measured from the same participants at the same time, the estimated structural paths and indirect effects are interpreted as associations consistent with the proposed pathway rather than evidence of temporal sequence or causal mediation.

The theoretical foundation of the study draws on the therapeutic landscape and restorative environment literatures. These perspectives suggest that physical, social, and aesthetic qualities of places may be associated with perceived therapeutic qualities and self-reported restoration (9, 17, 21, 41). Partial least squares structural equation modeling was used to test the research hypotheses. PLS-SEM is appropriate for explanatory and prediction-oriented models that include statistical mediation (42, 43). It enables direct and indirect associations among therapeutic landscape perception, restorative experience, satisfaction, and revisit intention to be estimated simultaneously.

### 3.2 Research area

The study was conducted in Palandöken Urban Forest in Erzurum Province. Located close to the city center on the foothills of Palandöken Mountain, the area is an important nature-based recreational setting for urban residents and visitors, offering opportunities for walking, relaxation, and picnicking. Although it is accessible from the city, its natural and mountainous landscape creates an environmental context that visitors may perceive as offering escape from daily life, contact with nature, mental relaxation, and restoration. Palandöken Urban Forest was therefore considered an appropriate site for examining associations among therapeutic landscape perception, restorative experience, satisfaction, and revisit intention. Its combination of urban accessibility and mountain landscape character provides a distinctive context, while the study does not assume that this setting produces objective health improvement. The location and general visual characteristics of the study area are presented in Figure 2.

**Figure 2:**
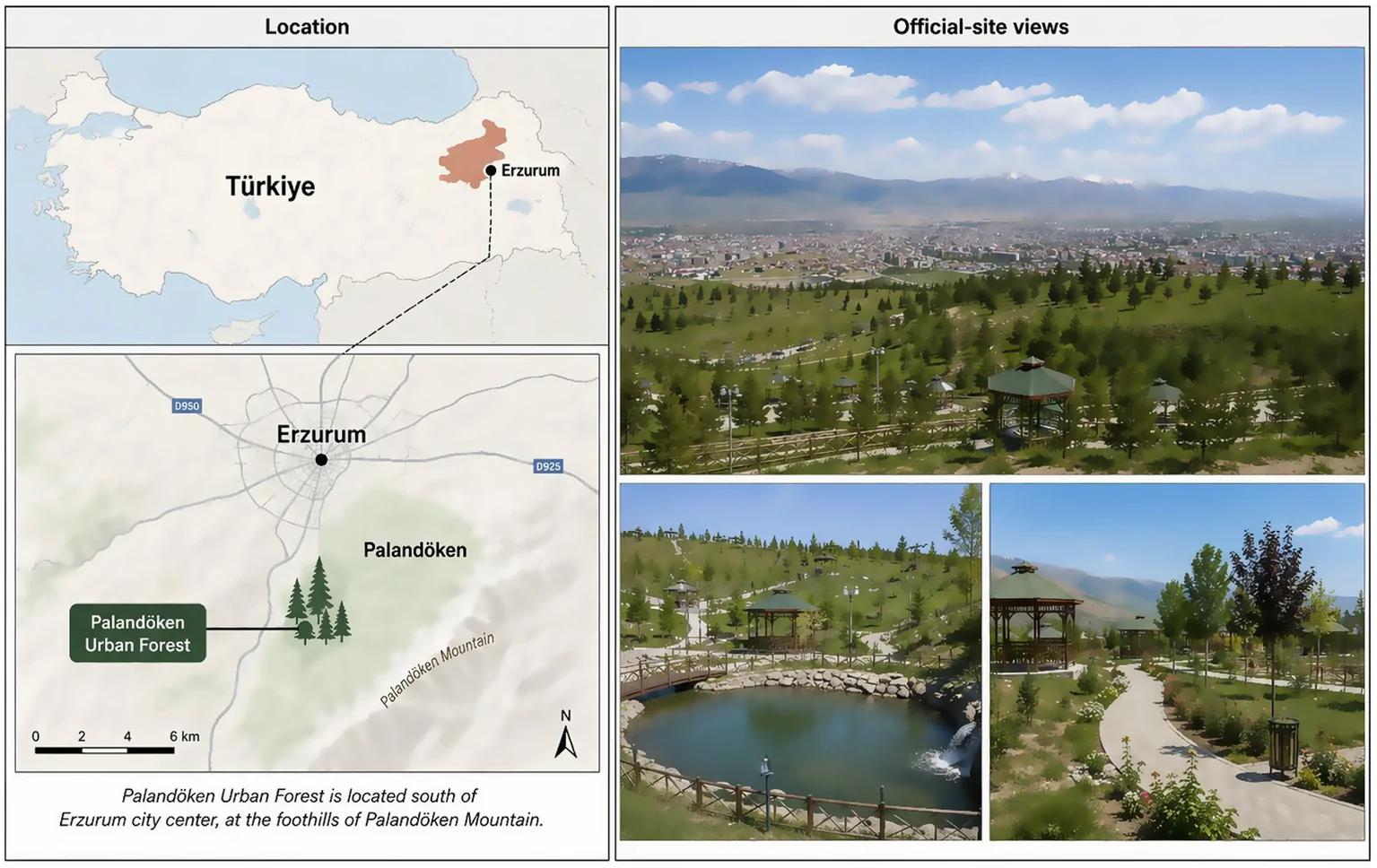
Study area: Palandöken Urban Forest, Erzurum, Türkiye. Source: Prepared by the authors based on official materials from Palandöken Municipality and the Erzurum Provincial Directorate of Culture and Tourism.

### 3.3 Population and sample of the study

The study population consisted of individuals who had visited Palandöken Urban Forest. Given the field conditions, visitor accessibility, and the collection of data within the natural flow of visits, a non-probability convenience sampling method was employed. The inclusion criteria were being 18 years of age or older, having visited Palandöken Urban Forest, and voluntarily agreeing to participate in the study.

Data were collected from visitors to Palandöken Urban Forest using a questionnaire. After incomplete, incorrectly completed, or inconsistent questionnaires had been excluded, the final analyses were based on 413 valid responses. Sample-size adequacy was evaluated using the criteria recommended for PLS-SEM. Model complexity, the maximum number of structural paths directed toward an endogenous construct, expected effect size, significance level, and statistical power were considered together. The PLS-SEM literature recommends that sample size should not be determined solely according to the traditional “10-times rule”; instead, more sensitive approaches based on power analysis should be used (42, 43).

The rationale for the sample size was established through a G*Power-based statistical power analysis using a multiple regression model. Revisit intention is the endogenous variable receiving the highest number of structural paths and is predicted by five variables: the three dimensions of therapeutic landscape perception, restorative experience, and satisfaction. In Cohen’s classification, *f*^2^ = 0.02 represents a small effect, *f*^2^ = 0.15 a medium effect, and *f*^2^ = 0.35 a large effect (44). These values are not absolute cut-off points but general guidelines for power analysis. Expected effect size should be justified by considering the research context, previous findings, and the minimum effect size intended to be detected (45–47). Because the relationships in the model may occur at a small-to-medium level and mediation effects may be relatively smaller, an *f*^2^ value of 0.05 was adopted as a conservative assumption. With the significance level set at *α* = 0.05, statistical power at 0.95, and the maximum number of predictors at 5, the minimum required sample size was approximately 402. The 413 valid questionnaires therefore exceeded the recommended minimum and were considered sufficient for PLS-SEM analysis.

### 3.4 Data collection instrument

A structured questionnaire was used as the data collection instrument. It consisted of two main sections. The first section included questions on participants’ demographic characteristics and visit behavior, including gender, age, educational status, income level, occupation, and visit frequency. The second section contained scale items measuring therapeutic landscape perception, restorative experience, visitor satisfaction, and revisit intention. The items were adapted from scales previously used and tested for validity in the relevant literature. All items were measured using a 5-point Likert-type scale ranging from 1 = Strongly disagree to 5 = Strongly agree. The scales were adapted from previous studies (Table 1) and modified to suit the context of Palandöken Urban Forest. Based on the theoretical framework of this study,

**Table 1:** Sources of the adapted scales.

Variable	Number of items	Scale source
Therapeutic landscape perception	11	Feng et al. (35), Breiby and Slåtten (59)
Restorative experience	8	Tang et al. (60)
Visitor Satisfaction	4	Rasoolimanesh et al. (61), Jiang et al. (62)
Revisit intention	3	Seyfi et al. (63)

The translation–back-translation method was followed when adapting the scales into Turkish. First, two researchers familiar with the field’s terminology translated the items from English into Turkish. An independent language expert who had not seen the original scales then translated the Turkish version back into English. The back-translated version was compared with the original items, and expressions involving semantic shifts, conceptual differences, or contextual inconsistencies were reviewed. The items were subsequently evaluated by a panel of three academic subject-matter experts with expertise directly relevant to the study (one professor and two associate professors) for linguistic clarity, conceptual equivalence, content relevance, and suitability for the context of Palandöken Urban Forest; minor revisions were made where necessary. This process aimed to ensure that the items were appropriate for the target sample linguistically, conceptually, and contextually. It is consistent with approaches recommending the combined use of translation–back-translation, expert evaluation, and conceptual equivalence checks in cross-cultural scale adaptation (48–50). Data were collected face to face from visitors to Palandöken Urban Forest between 04.06.2026 and 15.06.2026. Participants were informed about the purpose of the study, and participation was entirely voluntary.

### 3.5 Data analysis

The research data were analyzed in two stages. In the first stage, the data were examined for missing values, coding errors, outliers, response consistency, and basic distributional characteristics. Following these checks, the final data set was prepared for PLS-SEM analysis. In the second stage, the research model was tested using partial least squares structural equation modeling in SmartPLS 4.

The PLS-SEM analysis proceeded in two stages: assessment of the measurement model and assessment of the structural model. First, the reliability and validity of the constructs were examined; the hypothesized direct and indirect relationships were then tested. For the measurement model, indicator loadings, Cronbach’s alpha, rho_A, composite reliability, AVE, the Fornell–Larcker criterion, and HTMT values were evaluated. For the structural model, VIF values, path coefficients, *t*-values, *p*-values, *R*^2^, and *f*^2^ were examined. The significance of indirect effects was evaluated using bootstrap confidence intervals. Statistical significance was interpreted separately from substantive magnitude, particularly for paths with very small *f*^2^ values.

## 4 Findings

The findings are presented in accordance with the logic of PLS-SEM. The sample structure and item-level distributions are reported first, followed by common method bias checks, the measurement model, discriminant validity, the structural model, and mediation effects. This sequence establishes whether the model meets the measurement requirements before the theoretically proposed associations are evaluated.

### 4.1 Demographic characteristics of the participants and descriptive statistics of the measurement items

The profile of the 413 participants is presented in Table 2. The sample was relatively balanced by gender, with women accounting for 46.5% and men for 53.5% of participants. The largest age group was 25–34 years (27.1%), followed by the 35–44 age group (22.8%). The distributions of education and occupation indicate that Palandöken Urban Forest attracts visitors from diverse sociodemographic groups rather than a single user profile. Most participants were from Erzurum (77.7%), highlighting the area’s strong local urban-forest function.

**Table 2:** Participant profile.

Variable	Category	*n*	%
Gender	Female	192	46.5
	Male	221	53.5
	Total	413	100.0
Age	18–24	71	17.2
	25–34	112	27.1
	35–44	94	22.8
	45–54	74	17.9
	55 and above	62	15.0
	Total	413	100.0
Education level	Primary education	54	13.1
	High school	151	36.6
	Associate degree	61	14.8
	Bachelor’s degree	115	27.8
	Postgraduate degree	32	7.7
	Total	413	100.0
Occupation/employment status	Student	89	21.5
	Public sector employee	103	24.9
	Private sector employee	82	19.9
	Self-employed	67	16.2
	Retired	43	10.4
	Unemployed	29	7.0
	Total	413	100.0
Income level	Low	130	31.5
	Medium	248	60.0
	High	35	8.5
	Total	413	100.0
Visitor origin	From erzurum	321	77.7
	From outside erzurum	92	22.3
	Total	413	100.0
Frequency of visiting the area	First time	79	19.1
	Rarely	77	18.6
	Occasionally	127	30.8
	Frequently	81	19.6
	Regularly	49	11.9
	Total	413	100.0

The item means presented in Table 3 are generally high. Mean scores for the therapeutic landscape perception items range from 4.12 to 4.50, indicating positive perceptions of the area’s natural, aesthetic/symbolic, and social dimensions. The restorative experience items also have high mean values. These results indicate that visitors report their experience of Palandöken Urban Forest not only as a physical recreational activity but also as an experience involving escape, relaxation, and restoration. These self-reported perceptions should not be interpreted as objective evidence of health improvement.

**Table 3:** Descriptive statistics of the variables.

Construct	Dimension	Item	*N*	Mean	SD	Skewness	Kurtosis
Therapeutic landscape perception	Natural landscape	TLP1	413	4.50	0.67	−0.99	−0.22
		TLP2	413	4.38	0.73	−0.73	−0.79
		TLP3	413	4.34	0.76	−0.66	−0.99
	Aesthetic landscape	TLP8	413	4.27	0.79	−0.52	−1.21
		TLP9	413	4.43	0.69	−0.80	−0.55
		TLP10	413	4.35	0.74	−0.67	−0.90
		TLP11	413	4.29	0.78	−0.56	−1.15
	Social landscape	TLP4	413	4.18	0.84	−0.35	−1.50
		TLP5	413	4.12	0.87	−0.24	−1.64
		TLP6	413	4.37	0.73	−0.70	−0.83
		TLP7	413	4.23	0.82	−0.45	−1.37
Restorative experience		RE1	413	4.27	0.81	−0.53	−1.27
		RE2	413	4.22	0.85	−0.44	−1.48
		RE3	413	4.28	0.80	−0.55	−1.23
		RE4	413	4.40	0.71	−0.75	−0.69
		RE5	413	4.14	0.89	−0.28	−1.68
		RE6	413	4.31	0.78	−0.60	−1.11
		RE7	413	4.21	0.84	−0.41	−1.46
		RE8	413	4.10	0.91	−0.20	−1.77
Visitor satisfaction		VS1	413	4.32	0.68	−0.50	−0.79
		VS2	413	4.56	0.64	−1.15	0.19
		VS3	413	4.51	0.61	−0.85	−0.28
		VS4	413	4.42	0.69	−0.77	−0.60
Revisit intention		RI1	413	3.99	0.92	0.02	−1.82
		RI2	413	3.93	0.94	0.05	−1.70
		RI3	413	3.89	0.96	0.05	−1.61

### 4.2 Common method bias

Because the data were collected from the same participants using the same measurement instrument, common method bias was assessed using two complementary approaches. First, Harman’s single-factor test was conducted. The unrotated factor analysis showed that the first factor explained 22.71% of the total variance. As this value is well below the commonly used threshold of 50%, the results indicate that the variance cannot be attributed to a single dominant factor (51). Second, the full-collinearity VIF values recommended for PLS-SEM were examined. All VIF values were below the threshold of 3.3 recommended by Kock (52), indicating that common method bias was not at a level that would threaten the model results (Table 4).

**Table 4:** Full collinearity VIF results.

Construct	Full collinearity VIF
Natural landscape	1.466
Aesthetic landscape	1.481
Social landscape	1.278
Restorative experience	2.463
Visitor satisfaction	2.445
Revisit intention	2.120

### 4.3 Measurement model results

The measurement model was assessed in terms of internal consistency reliability, convergent validity, and discriminant validity (Figure 3).

**Figure 3:**
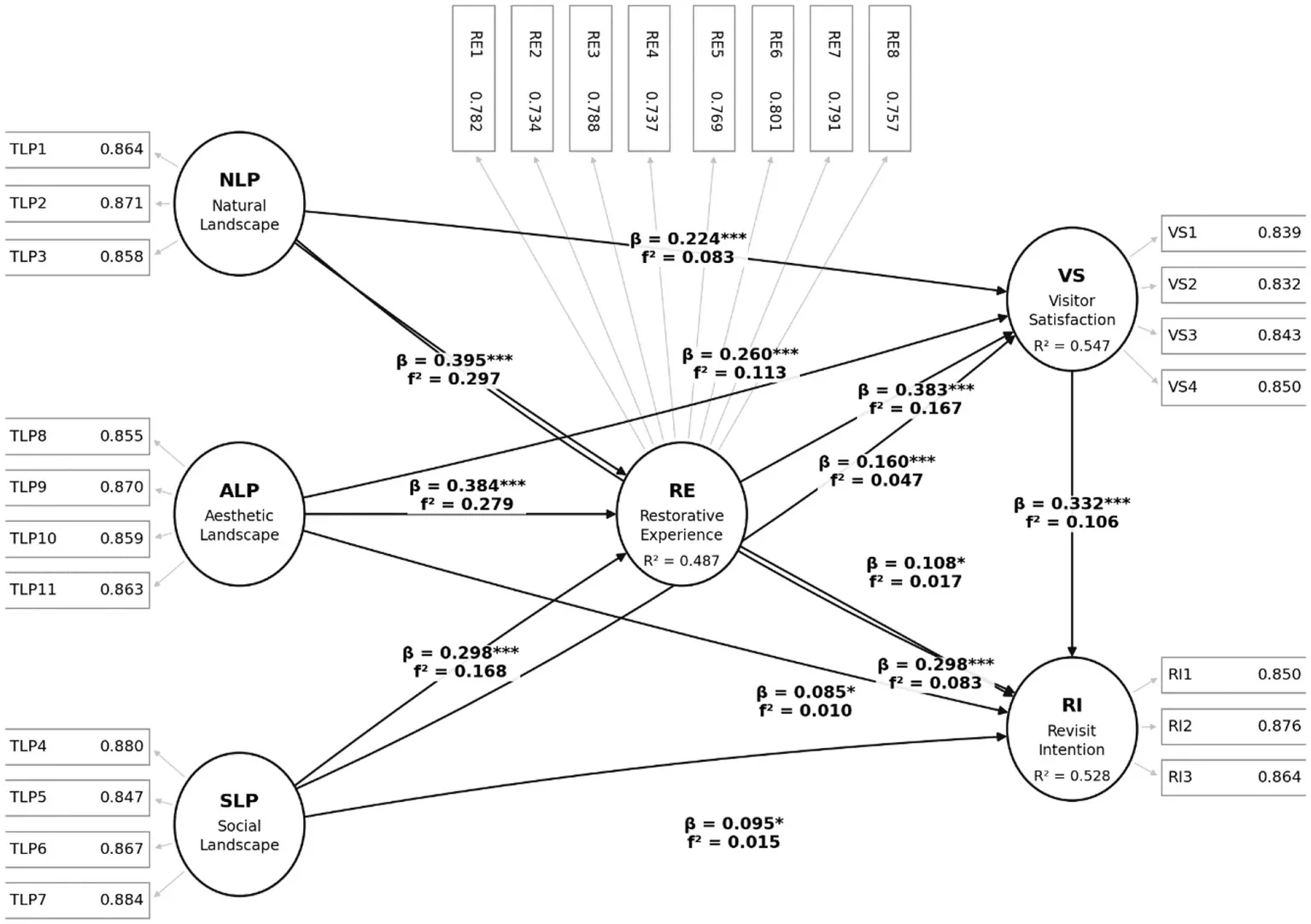
Reliability and validity.

The measurement model was evaluated by jointly considering indicator loadings, Cronbach’s alpha, rho_A, composite reliability, and AVE. In the PLS-SEM literature, indicator loadings should preferably exceed 0.70, composite reliability and Cronbach’s alpha should exceed 0.70, and AVE should exceed 0.50. These thresholds are considered sufficient for convergent validity and internal consistency (42). The results presented in Table 5 are consistent with these criteria.

**Table 5:** Measurement model results.

Construct/item	Factor loading
Natural landscape (*α* = 0.831; rho_A = 0.831; CR = 0.899; AVE = 0.747)	
TLP1/The clean air of Palandöken Urban Forest makes me feel better	0.864
TLP2/The climate and natural environment of Palandöken Urban Forest contribute to my physical and mental wellbeing.	0.871
TLP3/The natural/ecological environment of Palandöken Urban Forest has a positive effect on my health and wellbeing.	0.858
Aesthetic landscape (*α* = 0.885; rho_A = 0.888; CR = 0.920; AVE = 0.743)	
TLP8/The scenery and visual qualities of Palandöken Urban Forest make me feel relaxed.	0.855
TLP9/The mountain, forest, and open-space views of Palandöken Urban Forest are aesthetically impressive.	0.870
TLP10/The natural and visual atmosphere of Palandöken Urban Forest supports my sense of wellbeing.	0.859
TLP11/The landscape aesthetic value of Palandöken Urban Forest makes my visit experience meaningful.	0.863
Social landscape (*α* = 0.892; rho_A = 0.893; CR = 0.925; AVE = 0.756)	
TLP4/The services and use opportunities in Palandöken Urban Forest positively affect my visit experience.	0.880
TLP5/I feel safe in Palandöken Urban Forest.	0.847
TLP6 The social atmosphere in Palandöken Urban Forest is comfortable and peaceful.	0.867
TLP7 The interpersonal interaction/social atmosphere in Palandöken Urban Forest provides a positive visit experience.	0.884
Restorative experience (*α* = 0.902; rho_A = 0.903; CR = 0.921; AVE = 0.593)	
RE1/In Palandöken Urban Forest, I get away from the pressures of everyday life.	0.782
RE2/When I am in this area, I temporarily forget my daily problems.	0.734
RE3/Palandöken Urban Forest helps me reduce my stress.	0.788
RE4/I feel relaxed and free in this area.	0.737
RE5/In Palandöken Urban Forest, my mind recovers.	0.769
RE6/I feel more peaceful in this area.	0.801
RE7/In Palandöken Urban Forest, I feel renewed	0.791
RE8/This area gives me energy.	0.757
Visitor satisfaction (*α* = 0.862; rho_A = 0.863; CR = 0.906; AVE = 0.708)	
VS1/Overall, I am satisfied with my visit to Palandöken Urban Forest.	0.839
VS2/Palandöken Urban Forest met my expectations.	0.832
VS3/The time I spent in Palandöken Urban Forest was valuable and enjoyable.	0.843
VS4/I think my decision to visit Palandöken Urban Forest was right.	0.850
Revisit intention (*α* = 0.829; rho_A = 0.830; CR = 0.898; AVE = 0.745)	
RI1/I intend to revisit Palandöken Urban Forest in the future.	0.850
RI2/When I have the opportunity, I would choose Palandöken Urban Forest again.	0.876
RI3/It is highly likely that I will revisit Palandöken Urban Forest in the coming period.	0.864

For the three dimensions of therapeutic landscape perception, factor loadings ranged from 0.858 to 0.871 for natural landscape, from 0.855 to 0.870 for aesthetic landscape, and from 0.847 to 0.884 for social landscape. The loadings for restorative experience ranged from 0.734 to 0.801, while all loadings for visitor satisfaction and revisit intention exceeded 0.831. Thus, none of the indicators fell below the acceptable loading threshold. AVE values ranged from 0.593 to 0.756, and composite reliability values ranged from 0.898 to 0.925. These findings indicate that the items consistently represent their respective constructs and that the measurement model satisfies the requirements for convergent validity. The health-related wording appearing in individual scale items represents participants’ self-reported perceptions and is not treated as an objective clinical health measure.

### 4.4 Discriminant validity

Discriminant validity was assessed using the HTMT ratio and the Fornell–Larcker criterion. An HTMT value below 0.85 is used as a conservative threshold, whereas a value below 0.90 represents a more lenient criterion (42, 53). According to the Fornell–Larcker criterion, the square root of each construct’s AVE should exceed its correlations with the other constructs. The results presented in Table 6 indicate that discriminant validity was established according to both criteria.

**Table 6:** Discriminant validity analysis results (HTMT and Fornell–Larcker criterion).

HTMT	1	2	3	4	5	6
NLP	–	–	–	–	–	–
ALP	0.139	–	–	–	–	–
SLP	0.139	0.150	–	–	–	–
RE	0.548	0.527	0.441	–	–	–
VS	0.541	0.557	0.424	0.763	–	–
RI	0.511	0.479	0.420	0.750	0.778	–
Fornell–Larcker criterion	1	2	3	4	5	6
NLP	0.864	–	–	–	–	–
ALP	0.120	0.862	–	–	–	–
SLP	0.120	0.133	0.869	–	–	–
RE	0.477	0.471	0.396	0.770	–	–
VS	0.457	0.488	0.373	0.675	0.842	–
RI	0.424	0.413	0.361	0.651	0.659	0.863

The HTMT values range from 0.139 to 0.778 and remain below the critical thresholds. The highest HTMT value is observed between visitor satisfaction and revisit intention; nevertheless, it remains below 0.90. In the Fornell–Larcker results, the diagonal value for each construct is also higher than the correlations in the corresponding row and column. These results support statistical discriminant validity, although they do not by themselves eliminate concerns about conceptual or item-content overlap between therapeutic landscape perception and restorative experience.

### 4.5 Structural model results

The structural model was evaluated by first examining inner multicollinearity and then interpreting the path coefficients, *t*-values, *p*-values, and effect sizes. In PLS-SEM, VIF values below 5—and below 3.3 under more conservative criteria—indicate that multicollinearity is not critical. Following Cohen’s (44) classification, *f*^2^ values of approximately 0.02, 0.15, and 0.35 were interpreted as small, medium, and large effects, respectively. In two-tailed tests, a *t*-value above 1.96 indicates significance at the 5% level. Statistical significance and substantive magnitude were evaluated separately.

As shown in Table 7, the VIF values range from 1.026 to 2.275, indicating no multicollinearity problem in the structural model. All three dimensions of therapeutic landscape perception show statistically significant positive associations with restorative experience. Natural landscape (*β* = 0.395, *t* = 10.721), aesthetic landscape (*β* = 0.384, *t* = 10.438), and social landscape (*β* = 0.298, *t* = 7.763) are positively associated with restorative experience. Accordingly, H1a, H1b, and H1c were supported. These results are consistent with the proposition that naturalness, aesthetic meaning, and social atmosphere are jointly related to visitors’ self-reported restoration.

**Table 7:** Structural model results.

Path	InnerVIF	*β*	*t*	*p*	*f* ^2^	Result
NLP → RE	1.026	0.395	10.721	<0.001	0.297	Supported
ALP → RE	1.030	0.384	10.438	<0.001	0.279	Supported
SLP → RE	1.030	0.298	7.763	<0.001	0.168	Supported
NLP → VS	1.331	0.224	5.388	<0.001	0.083	Supported
ALP → VS	1.317	0.260	6.486	<0.001	0.113	Supported
SLP → VS	1.202	0.160	4.127	<0.001	0.047	Supported
RE → VS	1.950	0.383	8.166	<0.001	0.167	Supported
NLP → RI	1.442	0.108	2.411	0.016	0.017	Supported
ALP → RI	1.466	0.085	2.035	0.043	0.010	Supported
SLP → RI	1.259	0.095	2.472	0.014	0.015	Supported
RE → RI	2.275	0.298	5.547	<0.001	0.083	Supported
VS → RI	2.211	0.332	6.011	<0.001	0.106	Supported

A similar pattern is observed for satisfaction. The natural, aesthetic, and social landscape dimensions are positively associated with satisfaction, supporting H2a, H2b, and H2c. Restorative experience shows one of the stronger associations with satisfaction (*β* = 0.383, *t* = 8.166), thereby supporting H4. For revisit intention, restorative experience (*β* = 0.298, *t* = 5.547) and satisfaction (*β* = 0.332, *t* = 6.011) show more pronounced associations, supporting H5 and H6, respectively.

Although the direct paths from natural landscape (*β* = 0.108, *p* = 0.016, *f*^2^ = 0.017), aesthetic landscape (*β* = 0.085, *p* = 0.043, *f*^2^ = 0.010), and social landscape (*β* = 0.095, *p* = 0.014, *f*^2^ = 0.015) to revisit intention are statistically significant, all three effect sizes are below Cohen’s conventional small-effect benchmark of 0.02. Thus, H3a–H3c are statistically supported, but the substantive magnitude of these direct relationships is very weak. This distinction indicates that statistical significance should not be interpreted as evidence of a practically strong direct relationship.

The *R*^2^ values are 0.487 for restorative experience, 0.547 for visitor satisfaction, and 0.528 for revisit intention. These results indicate that the model explains a meaningful proportion of variance in the three endogenous constructs. Within the limits of the cross-sectional design, restorative experience and visitor satisfaction show stronger associations with revisit intention than the direct landscape-to-revisit paths (Figure 4).

**Figure 4:**
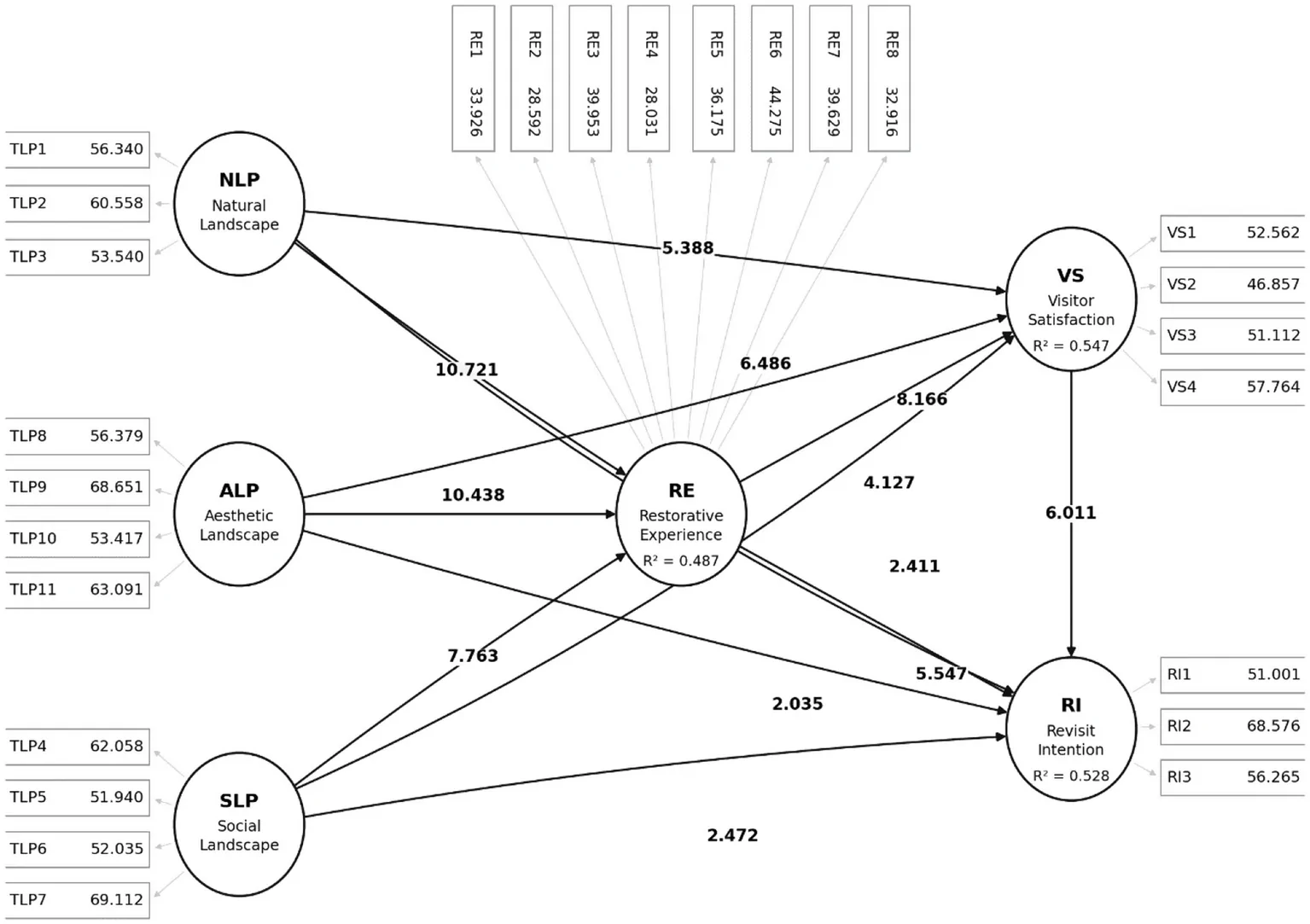
PLS-SEM results model.

### 4.6 Mediation effect results

The mediation analysis examines whether restorative experience accounts for statistically significant indirect associations between the therapeutic landscape dimensions and visitor outcomes. Specific indirect effects were evaluated using bootstrap confidence intervals. In PLS-SEM, an indirect effect is considered statistically significant when its bootstrap confidence interval does not include zero. This approach is regarded as more reliable than classical significance tests when indirect effects may not be normally distributed (54, 55). Because the data are cross-sectional, significant indirect effects are interpreted as associations consistent with the proposed mediation pathway rather than as evidence of temporal or causal mediation.

The results in Table 8 show statistically significant indirect associations between all therapeutic landscape dimensions and both visitor satisfaction and revisit intention through restorative experience. For satisfaction, the largest indirect coefficient is observed for natural landscape (*β* = 0.151), followed by aesthetic landscape (*β* = 0.147) and social landscape (*β* = 0.114). Accordingly, H7a, H7b, and H7c were supported. A similar pattern is observed for revisit intention: the indirect coefficients through restorative experience are *β* = 0.118 for natural landscape, *β* = 0.114 for aesthetic landscape, and *β* = 0.089 for social landscape. Accordingly, H8a, H8b, and H8c were supported. Because none of the confidence intervals includes zero, these indirect associations are statistically reliable and consistent with the proposed mediation model.

**Table 8:** Mediation effect results for restorative experience.

Mediation path	Indirect *β*	*t*	*p*	95% CI	VAF	Mediation interpretation
NLP → RE → VS	0.151	6.410	<0.001	0.108–0.203	40.3%	Partial
ALP → RE → VS	0.147	6.709	<0.001	0.107–0.190	36.2%	Partial
SLP → RE → VS	0.114	5.623	<0.001	0.076–0.153	41.7%	Partial
NLP → RE → RI	0.118	5.076	<0.001	0.070–0.164	52.2%	Partial
ALP → RE → RI	0.114	4.762	<0.001	0.067–0.165	57.5%	Partial
SLP → RE → RI	0.089	4.704	<0.001	0.053–0.127	48.2%	Partial

Because the corresponding direct paths also remain statistically significant, the pattern is conventionally classified as partial mediation. The VAF values range from 36.2 to 57.5%, indicating that a meaningful share of the modeled association is represented by the indirect path through restorative experience. These statistics do not establish that restorative experience temporally or causally transmits the effect of landscape perception. Rather, they indicate a cross-sectional pattern consistent with the proposed indirect pathway.

Taken together, the findings suggest that visitors may perceive Palandöken Urban Forest not merely as a recreational area but also as a public green space with therapeutic qualities. Perceptions of its natural, aesthetic, and social landscape components are associated with stronger self-reported restorative experiences, satisfaction, and revisit intention. This interpretation concerns perceived restoration and recreation-related outcomes and should not be taken as evidence of demonstrated clinical or objective health improvement.

To assess whether the mediation findings were sensitive to potential semantic overlap between selected therapeutic-landscape and restorative-experience indicators, additional sensitivity analyses were conducted using two alternative measurement specifications. In the first specification, RE4 and RE6 were excluded, while in the stricter second specification, RE3 was additionally excluded. Across both specifications, the reliability and validity of restorative experience remained satisfactory, the principal structural relationships retained their direction and significance, and all six specific indirect effects remained statistically significant. These findings indicate that the substantive conclusions of the study are robust to alternative measurement specifications and are not driven by the potentially overlapping indicators. Detailed results of the sensitivity analyses, including measurement-model statistics, structural path coefficients, explained variance, and bootstrap confidence intervals for the indirect effects, are presented in Tables A1 and A2.

## 5 Discussion

The results show that visitors perceive Palandöken Urban Forest not merely as a recreational space but also as an urban-forest setting associated with self-reported restoration and perceived wellbeing. The diversity of participants in terms of gender, age, education, occupation, and income indicates that the area serves a broad range of users. It functions as a shared nature-based public space used by diverse urban residents in everyday life. The predominance of participants from Erzurum further indicates that Palandöken Urban Forest is an accessible and valuable recreational resource, particularly for local users. The findings should therefore be interpreted not only as a tourist-destination experience but also as an urban green-space experience used regularly or periodically within daily life.

The descriptive findings further support this interpretation. High mean scores for therapeutic landscape perception, restorative experience, and visitor satisfaction indicate that visitors evaluate the area favorably in terms of its natural, aesthetic, and social qualities and report experiences involving escape, relaxation, mental relief, and renewal. These are self-reported perceptions and experiences, not objective indicators of health change. Although revisit intention is positive, it is lower than satisfaction. This suggests that, while visitors value the area and are satisfied with their experience, the decision to return may also depend on contextual factors such as season, weather, available time, transportation, alternative recreational opportunities, and personal schedules.

The structural model indicates that all three dimensions of therapeutic landscape perception are significantly associated with restorative experience. The strongest association is observed for natural landscape, which is consistent with the idea that forest structure, clean air, open views, silence, and perceptual distance from the city are related to reported mental relief and restoration. Schwartz et al. (56), for example, found that positive affect expressed during visits to urban parks in San Francisco increased and that the increase was more pronounced in greener park types. Similarly, a study of urban parks in the United States showed that expressed happiness was higher during park visits than during time spent outside parks; although the effect varied across cities, it generally indicated a strong urban-park benefit (57). These studies are compatible with the observed association between natural landscape perception and restorative experience in Palandöken, while the present study itself does not test pre-post psychological change.

The findings also indicate that restorative experience is associated with more than naturalness alone. The similar coefficients for aesthetic and natural landscapes suggest that scenery, mountain forms, seasonal atmosphere, visual attractiveness, and spatial spaciousness are also related to visitors’ self-reported restorative responses. This result is consistent with the therapeutic landscape framework, which integrates physical, social, aesthetic, and experiential elements. Bell et al. (17) emphasized that restorative experiences arise not only from physical surroundings but also from meaningful interactions established with places. The social landscape is also positively associated with restorative experience, although its coefficient is smaller than those of the natural and aesthetic dimensions. In public open spaces such as urban forests, the social dimension may reflect perceptions of safety, accessibility, organization, and tranquility rather than intense social interaction.

The study also identifies positive associations between the therapeutic landscape dimensions and satisfaction. This finding is consistent with Yao et al. (15), who showed that the recreational environment of urban forests is associated with visitor satisfaction and post-visit behavior. In an urban forest with a mountainous setting, scenery, aesthetic quality, and spatial character may be as relevant to visitor satisfaction as conventional facilities and accessibility.

Although the direct paths from the therapeutic landscape dimensions to revisit intention are statistically significant, their effect sizes are very small (*f*^2^ = 0.010–0.017). These relationships should therefore be interpreted as weak in substantive magnitude despite reaching statistical significance. The result is important because it separates statistical support for H3a–H3c from practical strength: favorable perceptions of natural, aesthetic, and social landscape qualities are related to revisit intention, but the direct associations are minimal. Restorative experience and satisfaction show comparatively stronger associations with revisit intention, suggesting that the modeled pathway is more informative when experiential and evaluative variables are considered alongside direct landscape perceptions.

Restorative experience occupies a central position in the proposed model and is positively associated with both visitor satisfaction and revisit intention. Visitors who report greater mental distance from daily pressures, relaxation, relief, and renewal also tend to report more favorable evaluations of the visit and stronger intentions to return. Li and He (29) similarly found that perceived restorative environments are associated with post-visit behavior through flow experience. In the Palandöken case, restorative experience can therefore be understood as an important experiential component of the proposed pathway, while the cross-sectional data do not establish that it causally produces satisfaction or revisit intention.

The positive association between satisfaction and revisit intention is consistent with established findings in tourism and recreation research. In this study, satisfaction is one of the strongest direct correlates of revisit intention. Yao et al. (15) reported a similar association in urban forests, while Zeng and Li (58) identified a relationship between satisfaction and the likelihood of revisiting and recommending mountain-based health-tourism experiences. Satisfaction and revisit intention remain recreation-related outcomes; neither is interpreted as a health outcome.

The indirect-effect results are consistent with the proposed statistical mediation model. The natural, aesthetic, and social landscape dimensions are associated with satisfaction and revisit intention both directly and indirectly through restorative experience. The higher VAF values for revisit intention indicate that the indirect component through restorative experience represents a considerable share of the modeled association. This finding parallels Li and He (29), who identified an indirect role for flow experience. The incremental theoretical insight of the present study lies in distinguishing environmental appraisal from self-reported restorative experience and showing that the direct landscape-to-revisit associations are substantively weak, whereas restorative and evaluative variables are more prominent within the modeled pattern. This is an explanatory refinement of an established framework rather than evidence of a newly established causal mechanism.

From a public-health perspective, visitor satisfaction and revisit intention should not be interpreted as health outcomes. Rather, they are recreation-related evaluative and behavioral outcomes that may indicate visitors’ willingness to continue using an urban green space associated with perceived restoration and wellbeing. The public-health relevance of the findings therefore concerns the potential role of accessible urban forests in supporting repeated engagement with environments that users perceive as restorative, rather than any demonstrated improvement in health status. This distinction connects public-space use and perceived restoration to public-health knowledge without equating behavioral intention with health improvement.

The findings should nevertheless be interpreted within their specific context. The comparatively stronger associations of natural and aesthetic landscapes with restorative experience may be related to Palandöken’s mountainous setting, high-altitude character, and capacity to create perceptual distance from urban life. The smaller but significant association of the social landscape may reflect the area’s public, local, and outdoor character. In other destination types particularly more commercial, indoor, service-intensive, or long-distance wellness and tourism settings the same pattern may differ.

### 5.1 Theoretical implications

The theoretical contribution of this study is best understood as an incremental refinement rather than the development of a new explanatory model. Its main value lies in empirically distinguishing therapeutic landscape perception from restorative experience and examining their different positions within the proposed framework. Therapeutic landscape perception represents visitors’ appraisal of the environmental qualities of the urban forest, whereas restorative experience represents a separate self-reported experiential state associated with those perceptions.

The findings add nuance to the relationship between environmental perception and revisit intention. Although the direct associations of the natural, aesthetic, and social landscape dimensions with revisit intention are statistically significant, their effect sizes are very small. By contrast, restorative experience and satisfaction show comparatively stronger associations with revisit intention, while the indirect effects through restorative experience are also statistically significant. This pattern suggests that favorable perceptions of landscape qualities alone have limited substantive relevance for revisit intention unless restorative and evaluative experiences are considered. The theoretical insight therefore lies less in proposing a new causal mechanism and more in clarifying the distinct explanatory roles of environmental appraisal, self-reported restoration, and recreation-related outcomes within the proposed pathway.

The public-health contribution of the study lies in extending understanding of how perceived qualities of urban green spaces are associated with self-reported restorative experiences and continued recreational engagement. Satisfaction and revisit intention are not treated as indicators of health improvement; instead, they help explain how favorable experiences are associated with visitors’ willingness to reuse publicly accessible green environments that they perceive as supportive of restoration and wellbeing.

### 5.2 Practical implications

The findings indicate that urban-forest planning should extend beyond the provision of physical facilities. In settings such as Palandöken Urban Forest, the quality of the experience is associated with visitor satisfaction and the likelihood of return. Management and planning may therefore prioritize interventions that protect the natural environment, strengthen appreciation of landscape and tranquility, and provide opportunities for visitors to gain mental distance from urban life.

The association between natural landscape perception and restorative experience highlights the value of preserving green areas, walking paths, clean air, open views, and quiet spaces for relaxation. The aesthetic dimension also indicates that Palandöken’s mountain character, seasonal changes, and scenic qualities are relevant to visitor experience. Viewpoints, seating and resting areas, photography points, and subtle design elements that reinforce local character may improve the recreational experience.

The social landscape dimension should likewise be considered. Visitors’ restorative experiences are associated not only with solitude but also with an environment perceived as safe, clean, well maintained, and tranquil. Directional signs, maintenance, perceived safety, crowd management, and the careful layout of seating and rest areas are therefore relevant to visitor satisfaction. These recommendations concern experiential and recreational quality; the present findings do not demonstrate clinical or objective health effects of such interventions.

## 6 Limitations

The results provide support for the proposed model but should be interpreted in light of several limitations. First, the study employed a cross-sectional design, and all focal variables were measured from the same participants at the same time. Accordingly, the structural paths and significant indirect effects establish associations consistent with the proposed model but do not establish temporal ordering, causal effects, or causal mediation. Longitudinal, experimental, or repeated-measures designs would be required to evaluate such claims more directly.

Second, the study did not assess objective health outcomes, clinical indicators, physiological measures, or pre-post changes in health status. The findings concern perceived therapeutic landscape qualities and self-reported restorative experiences. They should therefore not be interpreted as evidence that Palandöken Urban Forest produces demonstrated health improvement or clinical therapeutic effects.

Third, the data were collected at a single research site, Palandöken Urban Forest. Although this provides contextual depth, it limits the direct transferability of the findings to other urban forests. Convenience sampling constitutes another limitation. Although the sample size was adequate for the analysis, this method restricts claims that the sample represents the entire visitor population.

The study also relied on self-report measures. Although the common method bias checks indicated no critical problem, visitors reported their perceptions and intentions through the same questionnaire, and this limitation cannot be completely disregarded. Finally, the data collection period is particularly relevant in an area with a strong seasonal character such as Palandöken. Visitor experiences may vary between summer and winter, weekdays and weekends, and different weather conditions. An additional limitation concerns visitor origin and previous visitation frequency. Most respondents were residents of Erzurum, and only a relatively small proportion were first-time visitors. These characteristics may be independently associated with revisit intention, because local and repeat visitors may differ from non-local or first-time visitors in terms of familiarity with the area, accessibility, and opportunity to revisit.

## 7 Recommendations for future researchers

Future studies may test the model in different urban forests, urban parks, or mountain-based recreational areas to examine how the observed associations vary across contexts. Comparisons between high-altitude areas such as Palandöken and lower-altitude urban green spaces may reveal more clearly the environmental conditions under which perceived therapeutic qualities and restorative experiences are reported.

Seasonality also offers an important avenue for future research. Palandöken Urban Forest acquires different visual, climatic, and recreational characteristics in winter, spring, and summer, which may be associated with visitor perceptions and revisit intention. Future studies may therefore collect data across different seasons.

Longitudinal, experimental, or pre-post designs would be particularly valuable for establishing temporal ordering and examining whether changes in exposure to urban forests are followed by changes in restorative experience or health-related indicators. Where public-health claims are of interest, future research could incorporate objective or validated health measures, physiological indicators, or clinically relevant outcomes alongside self-reported perceptions.

The model may also be extended by testing the mediating or moderating roles of satisfaction, destination image, perceived value, place attachment, and e-WOM. Supporting quantitative findings with qualitative interviews may provide a more detailed understanding of why visitors perceive Palandöken Urban Forest as therapeutic or restorative. An additional limitation concerns visitor origin and previous visitation frequency. Most respondents were residents of Erzurum, and only a relatively small proportion were first-time visitors. These characteristics may be independently associated with revisit intention, because local and repeat visitors may differ from non-local or first-time visitors in terms of familiarity with the area, accessibility, and opportunity to revisit. Also, future studies should explicitly incorporate visitor origin and prior visitation frequency as control variables and examine whether the proposed relationships remain stable across local/non-local and first-time/repeat visitor groups.

Ethics Declaration: This study was approved by the Bingöl University Social and Human Sciences Scientific Research and Publication Ethics Committee (Decision No: E-79879538-044-2600042026) and was conducted in accordance with the principles of scientific research and publication ethics.

## 8 Conclusion

The results show that visitors perceive Palandöken Urban Forest not merely as a recreational space but also as an urban-forest setting associated with self-reported restoration and perceived wellbeing. The diversity of participants in terms of gender, age, education, occupation, and income indicates that the area serves a broad range of users. It functions as a shared nature-based public space used by diverse urban residents in everyday life. The predominance of participants from Erzurum further indicates that Palandöken Urban Forest is an accessible and valuable recreational resource, particularly for local users. The findings should therefore be interpreted not only as a tourist-destination experience but also as an urban green-space experience used regularly or periodically within daily life.

The descriptive findings further support this interpretation. High mean scores for therapeutic landscape perception, restorative experience, and visitor satisfaction indicate that visitors evaluate the area favorably in terms of its natural, aesthetic, and social qualities and report experiences involving escape, relaxation, mental relief, and renewal. These are self-reported perceptions and experiences, not objective indicators of health change. Although revisit intention is positive, it is lower than satisfaction. This suggests that, while visitors value the area and are satisfied with their experience, the decision to return may also depend on contextual factors such as season, weather, available time, transportation, alternative recreational opportunities, and personal schedules.

## Data Availability

The raw data supporting the conclusions of this article will be made available by the authors, without undue reservation.

## Appendix. Sensitivity analysis of alternative restorative-experience specifications

The original eight-item restorative-experience specification was retained as the primary model. Sensitivity Model 1 excluded RE4 and RE6 because these indicators showed the closest semantic correspondence with therapeutic-landscape indicators referring to relaxation and peacefulness. Sensitivity Model 2 additionally excluded RE3 as a stricter robustness check.

**Table A1.** Sensitivity analysis of alternative restorative-experience specifications.

Indicator/path	Original model	Sensitivity model 1	Sensitivity model 2
RE items retained	8	6	5
Cronbach’s *α*	0.902	0.874	0.851
Composite reliability (CR)	0.921	0.905	0.894
AVE	0.593	0.615	0.627
HTMT: NLP–RE	0.548	0.541	0.545
HTMT: ALP–RE	0.527	0.513	0.512
HTMT: SLP–RE	0.441	0.428	0.425
NLP → RE	0.395	0.387	0.386
ALP → RE	0.384	0.367	0.361
SLP → RE	0.298	0.284	0.276
RE → VS	0.383	0.367	0.328
RE → RI	0.298	0.272	0.251
*R*^2^ RE	0.487	0.454	0.442
*R*^2^ VS	0.547	0.546	0.533
*R*^2^ RI	0.528	0.524	0.520

Sensitivity Model 1 excludes RE4 and RE6. Sensitivity Model 2 excludes RE3, RE4, and RE6. The original eight-item restorative-experience specification remains the primary model.

Bootstrap 95% confidence intervals are reported in brackets; none of the intervals includes zero. These sensitivity results demonstrate robustness of the statistical indirect-effect pattern but do not alter the cross-sectional, noncausal interpretation of mediation.

**Table A2.** Specific indirect effects in the sensitivity models.

Specific indirect effect	Sensitivity model 1 *β* [95% CI]	Sensitivity model 2 *β* [95% CI]
NLP → RE → VS	0.142 [0.101, 0.187]	0.127 [0.086, 0.173]
ALP → RE → VS	0.135 [0.096, 0.178]	0.119 [0.080, 0.160]
SLP → RE → VS	0.104 [0.068, 0.146]	0.091 [0.056, 0.132]
NLP → RE → RI	0.105 [0.063, 0.154]	0.097 [0.056, 0.144]
ALP → RE → RI	0.100 [0.058, 0.147]	0.091 [0.053, 0.134]
SLP → RE → RI	0.077 [0.045, 0.115]	0.069 [0.039, 0.105]
